# Spontaneously retrievable knowledge of German general practitioners depending on time since graduation, measured with the progress test medicine

**DOI:** 10.3205/zma001342

**Published:** 2020-09-15

**Authors:** Michaela Zupanic, Jelena Kreuer, Daniel Bauer, Zineb M. Nouns, Jan P. Ehlers, Martin R. Fischer

**Affiliations:** 1 Witten/Herdecke University, Faculty of Health, Interprofessional and Collaborative didactics, Witten, Germany; 2 Clienia Schlössli AG, Klinik für Psychiatrie und Psychotherapie, Oetwil am See, Switzerland; 3 University of Bern, Faculty of Medicine, Institute for Medical Education (IML), Bern, Switzerland; 4 Helios Kliniken GmbH, Academy at Fresenius Helios, Berlin, Germany; 5 Witten/Herdecke University, Faculty of Health, Didactics and Educational Research in Health Science, Witten, Germany; 6 LMU Munich, University Hospital, Institute for Medical Education, Munich, Germany

**Keywords:** general practitioners, replication study, progress test medicine, knowledge development, life-long learning

## Abstract

**Background: **General practitioners and general internists occupy a key position in German and Austrian healthcare systems. They provide primary care and act as gatekeepers between medical disciplines and sectors of care. Their explicit medical knowledge levels, however, can be quite disparate.

**Objective: **This study analyses whether general practitioners’ performances on a standardised knowledge test changes with four relevant socio-demographic variables.

**Design: **The survey was based on the Progress Test Medicine (PTM), a standardised 200 item knowledge test on graduate level. After formal blueprinting and item analysis, 60 items of PTM were selected (“PTM-GP”) for our study.

**Participants:** PTM-GP was presented ad hoc to general practitioners and internists from Germany and Austria at a number of professional meetings in 2011. 161 volunteers completed the survey.

**Main measures:** For evaluation, correlation analysis (Spearman), Kruskal Wallis-tests for non-parametric data and an analysis of covariance (ANCOVA) were calculated.

**Results: **Overall, four indicators turned out to be slightly significant for the performance on the PTM-GP, namely:

1. time passed since graduation,
2. the grade received in the licensing examination,
3. the type of institution for postgraduate training, and
4. the medical specialisation.

**Conclusions: **Recent graduates performed better in the PTM-GP; a doctor’s licensing examination grade as well as training at a university hospital correlated positively with PTM-GP performance.

A general doctor’s knowledge level is moderately influenced by exam grades, time since graduation, the institutional affiliation of postgraduate training and medical specialisation. Individual changes in knowledge over time have to be deliberately considered in lifelong learning. In consequence, the on-going teaching of medical knowledge should be integrated mandatory and verifiable into general doctors’ everyday practices, e.g. through repetitive knowledge tests with individual feedback and recommendations for further continuing medical education (CME).

## Zusammenfassung

**Hintergrund: **Hausärzt*innen und Internist*innen nehmen eine Schlüsselposition im deutschen und österreichischen Gesundheitswesen ein. Sie sind für die Primärversorgung zuständig und fungieren als Gatekeeper zwischen den unterschiedlichen medizinischen Fach- und Versorgungsbereichen. Hinsichtlich ihres medizinischen Wissensstandes können sie sich jedoch deutlich unterscheiden.

**Zielsetzung:** Ziel dieser Studie ist die Beantwortung der Fragestellung, ob die Prüfungsleistungen von Hausärzt*innen in einem standardisierten Wissenstest von vier relevanten soziodemografischen Merkmalen beeinflusst werden.

**Material: **Der für die Studie verwendete Fragebogen basierte auf dem Progress Test Medizin (PTM), einem standardisierten Wissenstest auf Absolvierendenniveau bestehend aus 200 Fragen. Durch Berücksichtigung einer genauen Vorgabe zur Verteilung von klinischen Fächern und Organsystemen, dem sogenannten „Blueprint“, sowie nach einer Bewertung der Fragen, wurden 60 Fragen des PTM für diese Studie ausgewählt: PTM – General Practitioners („PTM-GP“).

**Teilnehmende: **Der PTM-GP wurde Hausärzt*innen und Internist*innen aus Deutschland und Österreich ad hoc im Rahmen von mehreren Fachtagungen im Jahr 2011 vorgelegt. Es nahmen 161 Freiwillige am Wissenstest teil.

**Methodik: **Zur Bewertung der Ergebnisse wurden Korrelationsanalysen (Spearman), Kruskal-Wallis-Tests für nicht-parametrische Daten sowie eine Kovarianzanalyse (ANCOVA) durchgeführt.

Ergebnisse: Insgesamt erwiesen sich vier Indikatoren als leicht signifikant für das Abschneiden beim PTM-GP:

1. die Zeit seit Studienabschluss,
2. die Note im 3. Abschnitt der Ärztlichen Prüfung,
3. die Art der Einrichtung, an der die Fachärzt*innenausbildung absolviert wurde, sowie
4. das medizinische Fachgebiet.

**Schlussfolgerung: **Kürzlich Graduierte schnitten beim PTM-GP besser ab; die Note im 3. Abschnitt der ärztlichen Prüfung sowie die Fachärzt*innenausbildung an einem Universitätsklinikum hatten einen positiven Einfluss auf die Prüfungsleistung im PTM-GP.

Der Wissensstand von Hausärzt*innen wird moderat beeinflusst von Examensnoten, der Zeit seit Studienabschluss, der institutionellen Zugehörigkeit in der postgradualen Fachärzt*innenausbildung und dem Fachgebiet. Individuelle Veränderungen des Wissensstandes im zeitlichen Verlauf müssen bei der Planung von Fortbildungsmaßnahmen (lebenslanges Lernen) sorgfältig berücksichtigt werden. Daher sollte eine kontinuierliche medizinische Fortbildung verpflichtend und nachprüfbar in den hausärztlichen Berufsalltag integriert werden, beispielsweise durch wiederholte Wissenstests mit individuellem Feedback und Empfehlungen für die ärztliche Fortbildung (CME).

## Introduction

General practitioners (GPs) are one of the central contact points for patients in the German and Austrian health care systems. They should have broad knowledge [1] in all medical disciplines to serve their role as a “gatekeeper” [2] in patient care. Driven by new technologies and procedures as well as a better exchange of information, available medical knowledge is increasing rapidly and doctors are required to continue their education during their practice in order to keep up with new developments. It is estimated that the half-life of medical knowledge is currently five to ten years [3], [4] and that medical knowledge as a whole approximately doubles every six to eight years [5]. Up-to-date medical knowledge is of high importance to general practitioners. Since the assessment of the current status of knowledge of primary care physicians has so far received little attention in Germany, our study aims to shed some light on the question as to what determines the medical knowledge of general practitioners and how their knowledge base changes over time. The publication “Changes over time in the knowledge base of practicing internists” by Ramsey [6] served as an inspiration and methodological model for the research question of our study. Ramsey identified several predictors of a good track record in knowledge-based exams. The procedure allowed a comparison of their results with the performance level of current graduates of that time. The results showed, among others findings, a significant negative correlation between the time elapsed since the medical licensing exam and the test performance; that the better the result of the original Internal Medicine Specialization Exam, the better the knowledge test performance; that internists trained at an academic hospital achieved significantly better results than colleagues who had their residency at non-academic hospitals; that, interestingly, there was no difference in the knowledge test result with regard to participation in further education courses; and that general internists achieved better test results than internists of technical sub-disciplines such as cardiologists or gastroenterologists [6].

Ramsey's results inspired the research questions underlying this work. Thus, we made the following assumptions for general practitioners in Germany and Austria:

1. The more years since graduation from medical school have passed, the less medical knowledge can be spontaneously called up in a knowledge test.
2. The better the grade in the final medical examination, the better the performance of GPs on a knowledge test.
3. GPs who started their postgraduate clinical training at university and teaching hospitals perform better on a knowledge test than those who were trained in hospitals without university affiliation.
4. The performance on a knowledge test concerning internal medicine depends on the specific specialist training of GPs.

With these assumptions, the study aims to address the research gap concerning GPs’ knowledge changes over time in Germany and Austria and at the same time looks for starting points for further development of continuing education and training for general practitioners.

## Subjects and methods

### Subjects

For this cross-sectional study, general practitioners were contacted during professional meetings between October 2010 and September 2011 at nine German quality circles as well as in Salzburg, Austria, during the annual conference of the German College of General Practitioners and Family Physicians (DEGAM). In total 161 participants, including 25 from Austria, completed the survey for evaluation. The statistically necessary minimum sample size was met for Kruskall-Wallis-tests (expected differences=7, power=0.8, n=112) and Spearman correlation analyses (correlation=0.3, power=0.8, n=84), but failed for analysis of covariance (ANCOVA) (covariates=2, power=0.8, n=269) (G*Power 3) [7]. The survey with overall 58 questions is divided into four sections:

1. socio-demographic information of study participants,
2. specialist training and additional training,
3. professional progression with career milestones and
4. CME (Continuing Medical Education) habits (see attachment 1 ).

After collecting the data, each participant received her/his personal results in comparison to the entire group as a feedback concerning the individual knowledge status by mail. 

#### Study instruments

Medical knowledge was assessed using a modified and shortened Progress Test Medicine (PTM). The original PTM of Charité Berlin consists of 200 Multiple Choice (MC)-questions from all medical specialties at German graduate level [8]. The three resulting PTM-GP-scores were “correct”, “false” and “total” (correct minus false). In contrast to common multiple choice tests, the PTM includes a “don’t know”- option”, which is supposed to reduce guessing when uncertain [9], [10]. The PTM no. 21 (Autumn 2009) served as basis for the “PTM for General Practitioners” (PTM-GP) with 60 MC-questions used in the present study. Using item analysis (based on data from graduate level test takers), the best items according to formal criteria with an item difficulty between P=.40-.80 [11] and an item total correlation r>.30 [12] were selected. Despite the reduction of items, there was no marked decrease in meaningfulness, since the items were selected respecting the original blueprint based on both organ systems and clinical subjects, and were examined for internal and external consistency. The number of items stating the subject distribution is shown in table 1 (Tab. 1). With 13 items the largest share was attributed to internal medicine topics.

#### Statistical analyses

The hypotheses that there are relationships between subjects’ socio-demographic variables (time since graduation, examination grade, residency, academic teaching hospital) and performance on PTM-GP Scores (correct, false and total) was investigated using correlation analyses (Spearman), with the correlation coefficient r as effect size. Kruskal-Wallis tests for non-parametric data were performed to prove that the middle ranks in the groups are the same. The test variable can be converted analogously into the effect size d [13], [14]. The influence of biographical variables on performance on PTM-GP total score was determined with analysis of covariance (ANCOVA) [15]. All statistical analyses were performed using SPSS 21.0 and R. The a-level was set at 5%.

## Results

### Demographic characteristics

The sample consisted of 161 participants (109 male, 67.7%) with an average age of 51 years (SD 8.4 years; range 31 to 67 years). The mean year of graduation was 1988, so that an average of 23 years had passed since graduation (SD 8.6 years; range 3 to 42 years). 125 participants (77.6%) reported their grade in the final licensing examination (missing n=36, 22.4%). 

89.4% (n=144) of the participants had completed their residency training. Completion was 16 years ago on average (SD 7.9 years; range 2 to 37 years). Training qualifications were as GP (Facharzt für Allgemeinmedizin in German; n=97, 60.2%), GP with internal medicine certification (n=21, 13.0%), internist (n=16, 9.9%) or family doctor (Praktischer Arzt in German; n=10, 6.2%). 

At the time of the survey, most participants (n=103, 64%) worked in a private practice, 37 (23%) in a non-academic teaching hospital, 14 (8.7%) in an academic teaching hospital and only 7 (4.3%) in a university hospital.^1^ The first employment was in a non-academic teaching hospital for most participants (n=69, 42.9%), 57 (35.4%) in an academic teaching hospital and 23 (n=14.3%) in a university hospital. 5 participants started their career in a private practice (3.1%; missing n=7, 4.3%). 

#### PTM-GP performance

The obtained PTM-GP-scores of the 161 participants were 50.0±5.1 correct responses (range 33-60), and 6.5±3.7 false responses (range 0-24) with the resulting mean test score of 43.5±8.0 (range 11-60). The more time passed since graduation, resp. year of graduation, the lower the score “correct” with a weak significant inverse correlation (r=-.166, p=.035) and the higher the score "false" with a weak significant positive correlation (r=.196, p=.013). Table 2 (Tab. 2) indicates the descriptive statistics (mean and standard deviation) in four categories, showing minor differences in time since graduation.

**Figure 1:**
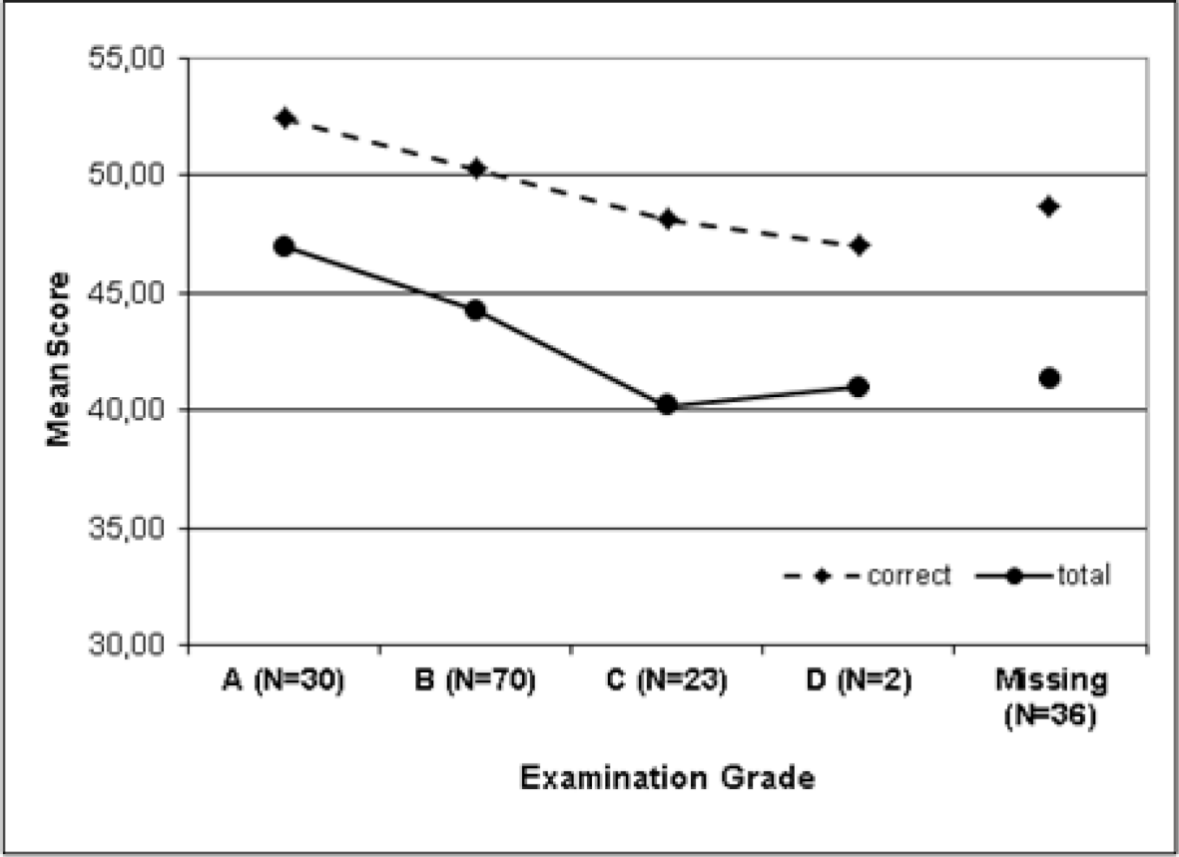
Examination grade and PTM-GP scores of participants (n=161)

The indicated examination grade of 125 participants was 2.0±0.7 (minimum 1, equivalent to A; maximum 4, equivalent to D). With a better score at graduation, correlation analyses yield fewer false answers (r=.188, p=.036) and significantly more correct answers (r=-.289, p=.001). The differences in PTM-GP scores depending on the examination grade is moderate significant for correct answers (Kruskal-Wallis test: χ^2^=9.5, p=.023, d=.382). Results for the five groups of participants, grade A (n=30, 18.6%), grade B (n=70, 43.5%), grade C (n=23, 14.3%), grade D (n=2, 1.2%), and missing (n=36, 22.4%) are shown in figure 1 (Fig. 1).

**Figure 2:**
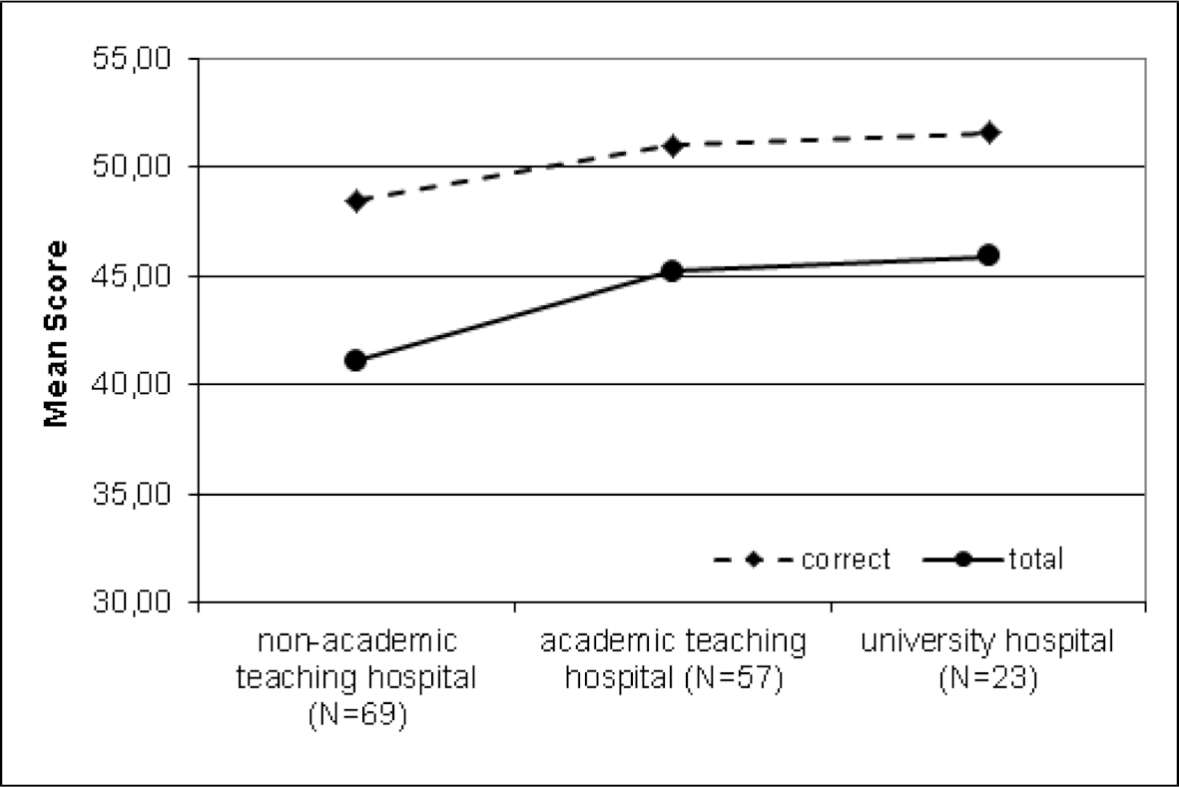
First employment and PTM-GP scores of participants (n=161; Missing 12 participants with n=5 in private practice and n=7 Missing)

In most participants (n=69, 42.9%), the first employment was in a non-academic teaching hospital, 57 (35.4%) in an academic teaching hospital and 23 (n=14.3%) in a university hospital (see figure 2 (Fig. 2)). These three groups were compared with a non-parametric Kruskal-Wallis test. The PTM-GP scores "correct" (χ^2^=8.8, p=.012, d=.424) and "total" are moderate significantly higher (χ^2^=7.9, p=.019, d=.394), when the first employment was at a university hospital (45.9±6.6, 95% CI 43.0-48.8) or academic teaching hospital (45.2±6.2, 95% CI 43.6-46.9), compared to first employment at non-academic teaching hospitals (41.1±9.3, 95% CI 38.9–43.3). However, there were no significant differences in results by taking the participants’ workplaces at the time of the survey (see above) into account (see figure 2 (Fig. 2)).

**Figure 3:**
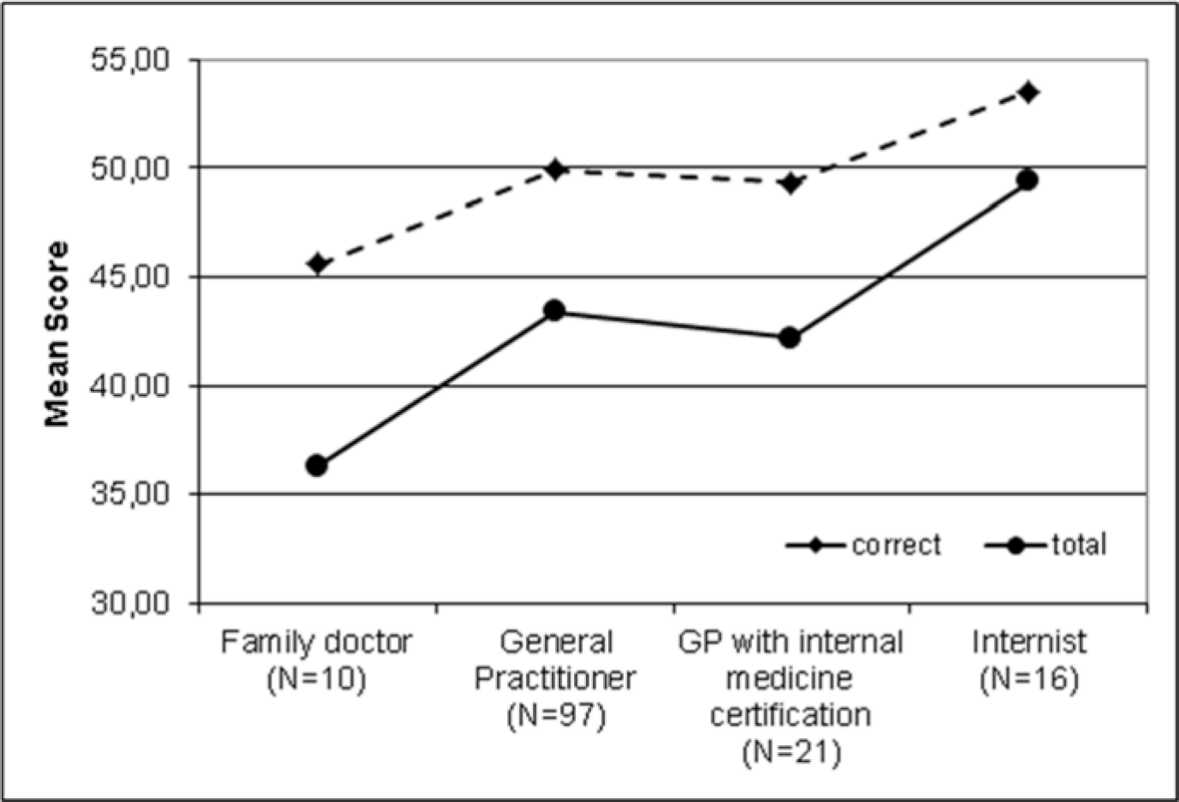
Specialist groups and PTM-GP scores of participants (n=161; Missing 17 participants not yet having completed residency training)

The PTM-GP scores of the four specialist groups general practitioner (n=97, 60.2%), general practitioner with internal medicine certification (n=21, 13.0%) and internists (n=16, 9.9%) or family doctor (n=10, 6.2%) differ moderately significant in non-parametric examination with Kruskal-Wallis test. This is true for “correct” answers (χ^2^=16.4, p=.001, d=.611), “false” answers (χ^2^=15.1, p=.002, d=.578) and the PTM-GP score "total" (χ^2^=16.9, p=.001, d=.623). Figure 3 (Fig. 3) shows that the best performance on the PTM-GP was achieved by the internists (49.4±8.4, 95% CI 44.9-53.8) and the lowest performance by the family doctors (36.3±7.8, 95% CI 30.7) (see figure 3 (Fig. 3)). 

Comparable results were found in the 13 questions on internal medicine. The PTM-GP scores of the four specialist groups were examined by using the non-parametric Kruskal-Wallis test. Moderate significant differences in the PTM-GP scores of the specialist groups for "correct" (χ^2^=11.3, p=.010, d=.473), “false” (χ^2^=13.4, p=.004, d=.533) and “total” (χ^2^=12.1, p=.007, d=.496) became clear and are shown in table 3 (Tab. 3).

To investigate the influence of the independent socio-demographic variables on the PTM-GP total score, an analysis of covariance (ANCOVA) was carried out with the specialist group and type of institution for postgraduate training, adjusted for time passed since graduation and the grade received in the licensing examination. When interpreting the results, it must be taken into account that the statistically necessary minimum sample for ANCOVA has not been reached (see above) and that 36 participants did not report their examination grade. Resulting sample for ANCOVA consist therefore of 105 participants (65% of the whole sample). Table 4 (Tab. 4) shows the results for the main effects of the within-groups factors and the covariates on the PTM-GP total score. The corrected model of ANCOVA explained moderate 27% of the variability (partial eta square=0.267). The examination grade had the greatest influence on performance (highly significant 10.8% clarification of variance) and time since graduation had a significant but lesser influence on PTM-GP total score (significant 6.8% clarification of variance). There is no effect of the two factors specialist group and residency, nor their interaction.

A further model of ANCOVA without the covariate examination grade and n=137 participants resulted in 19% explained variability (partial eta square=0.190). The greatest influence on performance had the type of residency (7.7% clarification of variance; significant). There were no other significant effects.

## Discussion

The assumptions of this replication study based on the results of the study by Ramsey can mostly be confirmed [6]. As shown in the results, there is a weak to moderate significant correlation between the spontaneously available general practitioner's knowledge and the

1. time elapsed since graduation,
2. the score in the licensing examination,
3. first career station and
4. specialist status.

### Time that has passed since the graduation 

The slight decrease of medical knowledge in the course of working life could be due to the fact that older GPs are less confronted with the patient problems of university hospital level in their daily work. In addition, it is likely they are less familiar with multiple-choice questions as their younger colleagues. This would mean that the exam questions do not represent a GP’s everyday routine well [16]. 

The “don’t know” option could also have caused a distortion. This option is included in the progress test medicine to discourage guessing and to simulate authentic clinical practice [17]. In the present study, 36 participants (22%) never chose the “don’t know” option. It may be that the “don’t know” option was ruled out, as it is an audit without real consequence (that is, without real decision-making situations on the patient with potentially threatening consequences), prompting bolder decisions from participants then they would have shown with real patients.

Carline [18], in contrast to Ramsey [6] and Norcini [19], found no significant correlation between the times elapsed since certification as an internist and the completion of a recertification test for internists [18]. However, they only examined physicians who had passed their first certification exam 5-10 years earlier. Due to the limitations mentioned, the results of Ramsey in 1991 and Norcini in 1985 may be considered more meaningful [6], [16]. Also, a multistage study from Canada comparing different methods of assessing primary care physicians found that medical literacy was inversely and significantly related to age and time since the exam [20]. 

#### Final grade in the licensing examination 

In the literature, the importance of exam grades for further degrees occupies a wide space. Ramsey showed that physicians with a higher examination grade performed better at the specialist examination, too [6]. In a German study it was found that the performance on the internist part of the progress test medicine in 2010 correlated significantly with the school leaving examination grade and the first part of the medical examination grade [21]. A similar correlation between the final grade and the examinations in the course of studies was demonstrated in numerous other studies [22], [23], [24]. 

The relationship between the level of knowledge and the completion of the specialist recertification examination and further examinations before physicians entered medical practice is described in several studies. In addition to school grades, other studies found influences of the country of origin, the field of study, the time between school and study achievement and the study section as relevant predictors [25], [26]. The obtained exam grade seems a good predictor for later performance on standard knowledge-based tests such as the knowledge test used in this study. In the model of ANCOVA, the examination grade had the greatest influence on PTM-GP performance and explained 10.8% of variance.

#### First career station 

The results showed that physicians who chose to continue education at a university hospital at the beginning of their careers achieved significantly better results in the knowledge test than physicians in non-academic hospitals. The nature of the first training facility, in turn, correlated with the score in the test score, as well as within the individual score groups. The extent of university affiliation of the first employment explains 7.7% of variance on PTM-GP performance, when the best predictor “exam grade” is not taken into account in the ANCOVA because of the many missing data points. It can be assumed that physicians at university hospitals are more likely to be involved in research and teaching and that their knowledge will be updated more reliably. This also has an impact on answering knowledge questions in tests. Due to the large teams of university hospitals, younger physicians are more challenged to justify themselves to their peers and to be more frequently audited in hierarchical structures. The more frequent “exam situation” trains quick-to-learn knowledge and may lead to better results in tests. 

It remains unclear whether the different training and further education on offer at training institutions has an impact on the level of knowledge. A study by Lehmann and Schultz found no significant difference in training behavior between physicians working in university hospitals and physicians working in peripheral care [27]. At university hospitals it takes longer to obtain the status of a specialist than that of houses with standard care, which is attributed, among other things, to the higher time spent on research and teaching [28]. Haffner and Schmidt postulate the creation of a training regulation that is based on the realistic competencies of future general practitioners and promotes their acquisition of medical knowledge. This is in their view the only way to strengthen the outpatient sector [29].

#### Specialist status

Specialists in internal medicine on average score more points in the test score of the knowledge test than general practitioners. One reason for this might be that practitioners are versed in a broad routine, but less well-prepared for specialized theoretical questions in the knowledge test. Another reason may be a sample matching age-based confounding effect, as more than 60% of the family doctors are among the older ones in the sample (median 52 years), whereas more than 60% of the internists belong to the younger participants. In the model of ANCOVA, the time since graduation resp. inverse age of participants explained significant 6.8% of the variance. Ramsey showed a rather opposite result than that found in this study [6]. According to their research, the well-educated generalists performed better than the specialized cardiologists and gastroenterologists. The contradiction is certainly also related to the different specialist training in Germany and the US.

#### Limitations of the current study 

While the comparison with data from the Federal Register of Physicians [30] shows a similar distribution pattern nationwide of general practitioners in terms of gender, age and specialist training, the recruitment of study participants to quality circles and congresses could have led to a selection bias, since it can be assumed that especially motivated, inquisitive general practitioners attend congresses and quality circles. Although all primary care physicians are required to undergo training and to collect CME points, a distortion of the study results cannot be ruled out. A randomized random sample was not possible in this study design. Possible effects due to the selection of participants are therefore not excluded. The motivation factor gains additional weight because the participants had to invest a relatively long time in answering the questionnaire with a scope of seven pages and the 60 test questions. Participation in the study was voluntary, reward or sanction was not expected in any case. An incentive may have been the offer of feedback concerning one’s individual knowledge status. But this is not comparable to a summative assessment for an academic and/or professional qualification, so that the lower motivation of the participants could have affected the results of the study. Ramsey found a similar limitation when comparing the results of voluntary study participants with the results of those physicians who had to undergo recertification and had a marked different degree of success [6]. A study focusing on low-stake tests, i. e. non-impact testing, may support the assumption that the external motivation or demotivation factors of the subjects could have played a role in the present study [31].

## Conclusion and outlook

Study results from the US [6] could be replicated in Germany and Austria although general practitioner’s education and career paths in the US and Germany are not directly comparable. The decline of medical knowledge over time seems to be relatively independent of the respective health care system.

This study was able to assess the performance of GPs in a standardized progress test and presents different predictors for their performance. In total, four indicators proved to be weak to moderate significant for performance:

1. The time elapsed since the licensing exam. There was a weak negative correlation between the time elapsed since graduation and the results of the knowledge test.
2. The grade achieved in the licensing exam. The results of the study show a moderate correlation between a good final grade and a good result in the knowledge test. The exam grade showed the greatest effect of the used socio-demographic variables on performance in ANCOVA.
3. The type of hospital in which the specialist training took place. Doctors who opted for continuing education in a university hospital at the beginning of their careers achieved moderate significantly better results in the knowledge test than doctors in non-academic hospitals did.
4. Whether the participant is a general practitioner or an internist. Internists achieved moderate better results on the knowledge test than general practitioners did.

While factual knowledge is a very important resource in the working life of a general practitioner, the experience-based and practical competence of a physician or the attention paid to the patient can be equally important. In order to keep it at the highest possible level over the entire period of active practice, the teaching of medical knowledge should be integrated into the non-academic everyday life of the general practitioner in a contemporary way, e.g. through repetitive progress tests with feedback on the individual knowledge profiles for planning their own further continuing medical education.

## Note

We chose not to exclude the 58 participants from the study who reported having their current primary workplace in inpatient care, because their attendance at quality groups aimed at GPs or their participation at the annual conference of the German College of General Practitioners and Family Physicians (DEGAM) respectively, suggested a mutual professional interest in primary care medicine, suggesting that albeit having different workplace settings they shared mutual patient cohorts.

## Acknowledgements

The authors thank all general practitioners who participated in this study and we owe sincere gratitude to the President of the DEGAM conference in Salzburg, Mr. Univ.-Prof. Dr. Andreas Sönnichsen, for supporting the implementation of this research. We would also like to thank Prof. Dr. med. Jörg Schelling and Mr. Matthias Holzer for supporting our study. 

## Competing interests

The authors declare that they have no competing interests. 

## Supplementary Material

Additional questionnaire for the Progress Test Medicine

## Figures and Tables

**Table 1:**
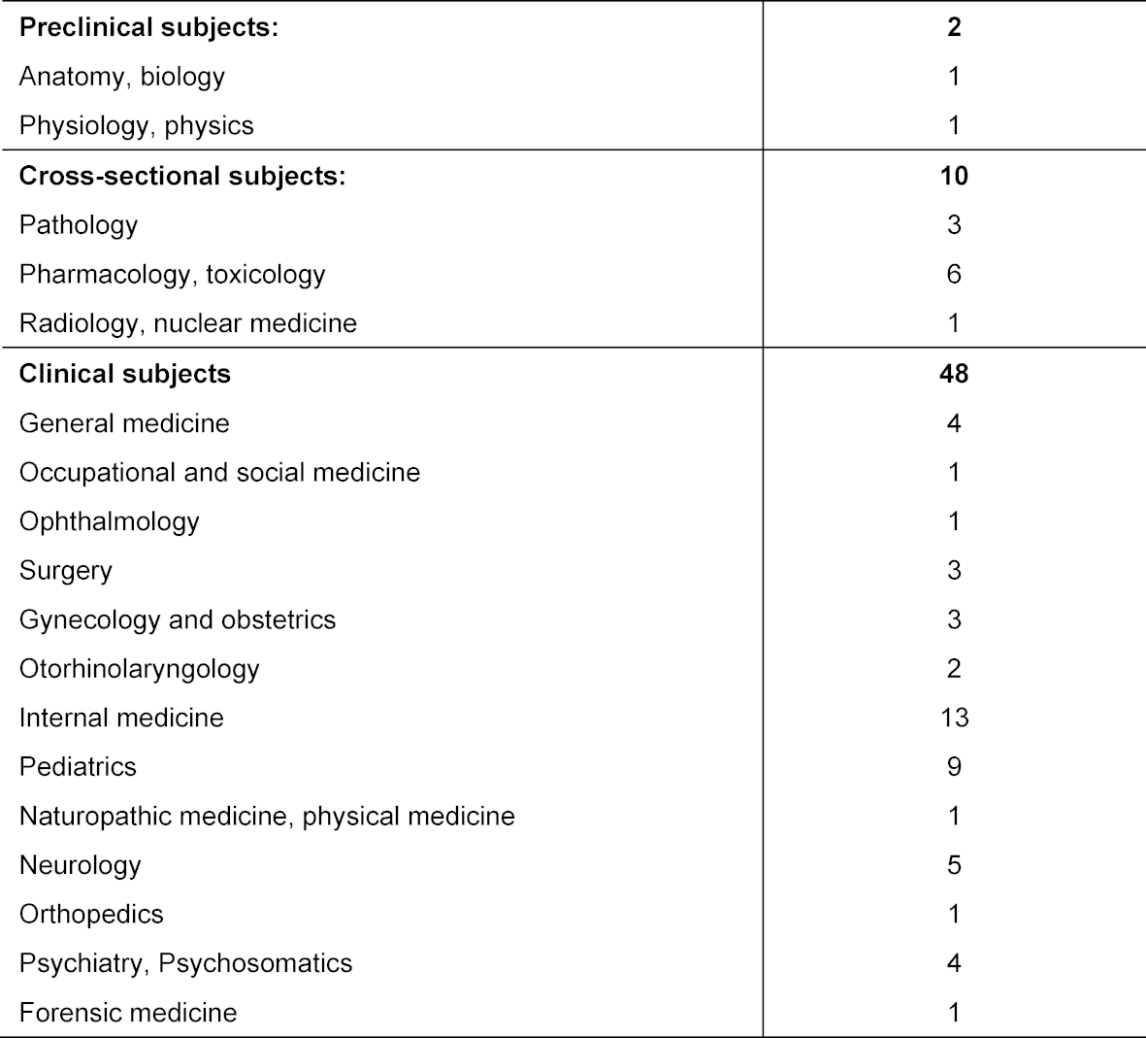
Number of items stating the subject distribution of PTM-GP(n=60)

**Table 2:**
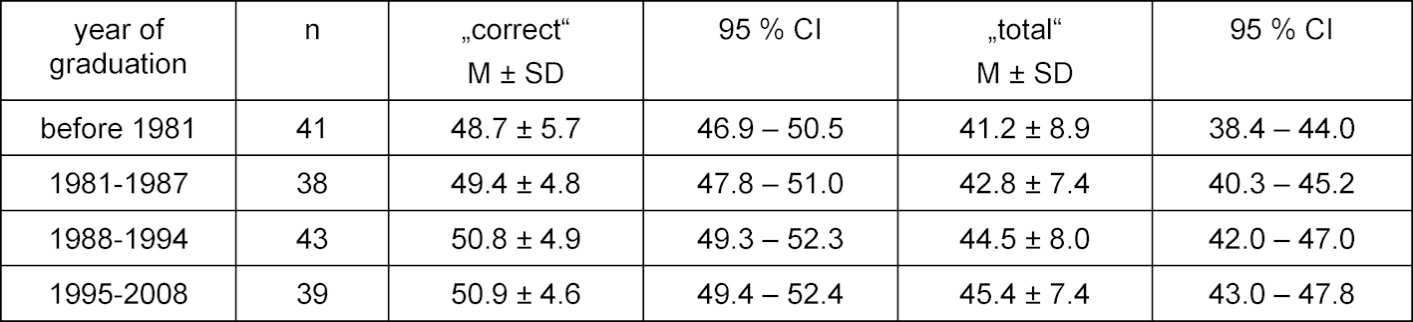
Categories regarding time since graduation and results of PTM-GP (n=161)

**Table 3:**
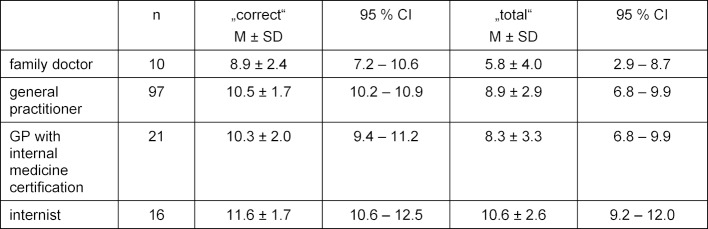
Specialist groups and results of PTM-GP concerning internal medicine (n=161)

**Table 4:**
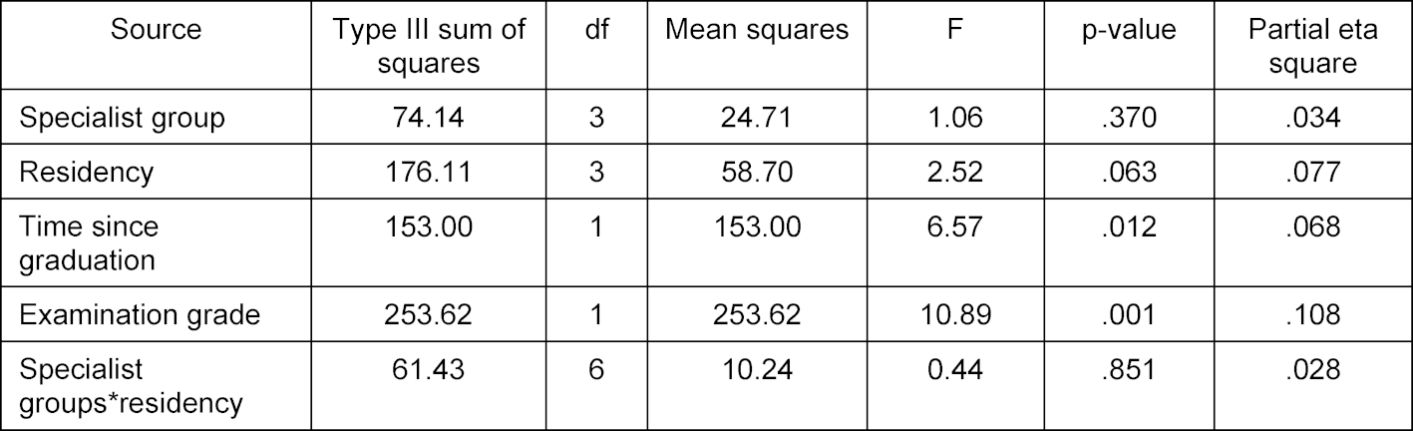
Socio-demographic variables and results of PTM-GP total Score in ANCOVA (n=105)
