# Supplementary material for: Spontaneously retrievable knowledge of German general practitioners depending on time since graduation, measured with the progress test medicine
Source: GMS J Med Educ. 2020 Sep 15;37(5):Doc49. doi: 10.3205/zma001342 (PMC7499467; doi:10.3205/zma001342)
Supplement: Additional questionnaire for the Progress Test Medicine [file JME-37-49-s-001.pdf]

Additional Questionnaire for the Progress Test Medicine

Dear participant,

this sheet is evaluated by machine. Please mark an answer in the following way: ⊗.

If you want to correct an answer, please fill in the incorrectly marked circle and beyond, like this: ● ⊗.

**Code (code of the PT answer sheet, only the last 5 digits!): (please enter below this line)**

Personal Information

**Gender**	O female O male
**Year of birth (please enter below this line):**	

Information on the course of study

**Beginning of medical studies in 19... (please enter below this line):**	
**Completion of medical studies in the year (please enter below this line):**	
**Grade in the 3rd state exam / final licensing examination (please enter below this line):**	
**Do you come from a medical family?**	O no O yes (parents)<br>O yes, others:
**Study in (place of study): ...... and duration: ..... (please enter below this line)**	
**Study in (place of study): ...... and duration: ..... (please enter below this line)**	
**Study in (place of study): ...... and duration: ..... (please enter below this line)**	
**Practical year or equivalent abroad:**	
Internal Medicine in (please enter below this line):	
Surgery in (please enter below this line):	
Elective subject (please include optional subject) in (please enter below this line):	
**Stays abroad:**	
Semester abroad - please specify the respective countries and length of stay:	
Internships - please indicate the respective countries and length of stay:	
Work - please indicate the respective countries and length of stay:	

Attachment 1 to: *Zupanic M, Kreuer J, Bauer D, Nouns ZM, Ehlers JP, Fischer MR. Spontaneously retrievable knowledge of Germany general practitioners depending on time since graduation, measured with the progress test medicine.* GMS J Med Educ. 2020;37(5):Doc49. DOI: 10.3205/zma001342

Residency training and additional training

**If you are still in residency training, please enter the subject and previous duration (in years) below this line.**		
**If you have already completed residency training, please enter the subject and year of completion below this line.**		
**Additional training (e.g. cardiology, neuropediatrics) completed in:**		
**Still in additional training in:**		
**General Practitioner / Internal Medicine:**		
O Family doctor O General practitioner O General practitioner with internal medicine certification O Internist		
**Other medical qualifications (e.g. sports medicine, TCM, naturopathy). Please indicate whether completed or still in training.**		
**Complementary medicine work on the patient:**		
Anthroposophic medicine	O 1 patient/day<br>O 1 patient/month	O 1 patient/week
TCM	O 1 patient/day<br>O 1 patient/month	O 1 patient/week
Naturopathy	O 1 patient/day<br>O 1 patient/month	O 1 patient/week
Homeopathy	O 1 patient/day<br>O 1 patient/month	O 1 patient/week
Other (Please also enter patient volume):		
**Graduation (PhD)**	O yes<br>O still in process	O no
**Publications in medical journals**	O none<br>O 6 to 10	O 1 to 5<br>O over 10
**Habilitation**	O yes<br>O still in process	O no
**Work in education, continuing education and training**		
O none O medical students O other health professionals (speech therapy, care...)<br>O continuing education and training of physicians O patient training<br>O non-medical education and training		

Attachment 1 to: *Zupanic M, Kreuer J, Bauer D, Nouns ZM, Ehlers JP, Fischer MR. Spontaneously retrievable knowledge of Germany general practitioners depending on time since graduation, measured with the progress test medicine.* GMS J Med Educ. 2020;37(5):Doc49. DOI: 10.3205/zma001342

Career path - important stages in your medical career

1. job (only jobs of at least 1 year)

**Subject:**	
O Internal medicine O General medicine<br>O other subject: ______________________________	
**Duration (please enter below this line):**	
**In:**	
O Germany O Austria O Switzerland<br>O other: ______________________________	
**When working in a clinic:**<br>(please check)	
**Is the clinic academically connected?**	
O university hospital O academic teaching hospital O non-academic teaching hospital	
**Care level of the clinic**	
O basic care O standard care O priority care O maximum care	
**Catchment area**	
O rather urban O rather rural	
**Rank**	
O doctor in internschip O assistent in continuing education O assistent in specalist status O Senior physician in charge<br>O Senior physician / Head O Chief physician	
**Current job**	O yes O no
**When working in a practice:**<br>(please check)	
**Academically connected practice?**	
O Teaching practice O unrelated practice	
**Organization type of practice**	
O practice without a partner O joint practice O practice sharing O medical care unit	
**Catchment area**	
O rather urban O rather rural	
**When working with partners or in a medical care unit**	
**Number of colleagues:**	O 1 O 2 O 3 O 4 O 5 O 6<br>O 7 O 8 O 9 O 10 O 11 O 12
**Subjects (subject, specialization) of the practice colleagues:**	
**Current job**	O yes O no

Attachment 1 to: *Zupanic M, Kreuer J, Bauer D, Nouns ZM, Ehlers JP, Fischer MR. Spontaneously retrievable knowledge of Germany general practitioners depending on time since graduation, measured with the progress test medicine.* GMS J Med Educ. 2020;37(5):Doc49. DOI: 10.3205/zma001342

2. job (only jobs of at least 1 year)

**Subject:**	
O Internal medicine O General medicine<br>O other subject: ____________	
**Duration (please enter below this line):**	
**In:**	
O Germany O Austria O Switzerland<br>O other: ____________	
**When working in a clinic:**<br>(please check)	
**Is the clinic academically connected?**	
O university hospital O academic teaching hospital O non-academic teaching hospital	
**Care level of the clinic**	
O basic care O standard care O priority care O maximum care	
**Catchment area**	
O rather urban O rather rural	
**Rank**	
O doctor in internschip O assistent in continuing education O assistent in specalist status O Senior physician in charge<br>O Senior physician / Head O Chief physician	
**Current job**	O yes O no
**When working in a practice:**<br>(please check)	
**Academically connected practice?**	
O Teaching practice O unrelated practice	
**Organization type of practice**	
O practice without a partner O joint practice O practice sharing O medical care unit	
**Catchment area**	
O rather urban O rather rural	
**When working with partners or in a medical care unit**	
**Number of colleagues:**	O 1 O 2 O 3 O 4 O 5 O 6<br>O 7 O 8 O 9 O 10 O 11 O 12
**Subjects (subject, specialization) of the practice colleagues:**	
**Current job**	O yes O no

Attachment 1 to: *Zupanic M, Kreuer J, Bauer D, Nouns ZM, Ehlers JP, Fischer MR. Spontaneously retrievable knowledge of Germany general practitioners depending on time since graduation, measured with the progress test medicine.* GMS J Med Educ. 2020;37(5):Doc49. DOI: 10.3205/zma001342

3. job (only jobs of at least 1 year)

**Subject:**	
O Internal medicine O General medicine<br>O other subject: ______________________________	
**Duration (please enter below this line):**	
**In:**	
O Germany O Austria O Switzerland<br>O other: ______________________________	
**When working in a clinic:**<br>(please check)	
**Is the clinic academically connected?**	
O university hospital O academic teaching hospital O non-academic teaching hospital	
**Care level of the clinic**	
O basic care O standard care O priority care O maximum care	
**Catchment area**	
O rather urban O rather rural	
**Rank**	
O doctor in internschip O assistent in continuing education O assistent in specalist status O Senior physician in charge<br>O Senior physician / Head O Chief physician	
**Current job**	O yes O no
**When working in a practice:**<br>(please check)	
**Academically connected practice?**	
O Teaching practice O unrelated practice	
**Organization type of practice**	
O practice without a partner O joint practice O practice sharing O medical care unit	
**Catchment area**	
O rather urban O rather rural	
**When working with partners or in a medical care unit**	
**Number of colleagues:**	O 1 O 2 O 3 O 4 O 5 O 6<br>O 7 O 8 O 9 O 10 O 11 O 12
**Subjects (subject, specialization) of the practice colleagues:**	
**Current job**	O yes O no

Attachment 1 to: *Zupanic M, Kreuer J, Bauer D, Nouns ZM, Ehlers JP, Fischer MR. Spontaneously retrievable knowledge of Germany general practitioners depending on time since graduation, measured with the progress test medicine.* GMS J Med Educ. 2020;37(5):Doc49. DOI: 10.3205/zma001342

4. job (only jobs of at least 1 year)

**Subject:**		
O Internal medicine O General medicine<br>O other subject: ____________________		
**Duration (please enter below this line):**		
**In:**		
O Germany O Austria O Switzerland<br>O other: ____________________		
**When working in a clinic:**<br>(please check)		
**Is the clinic academically connected?**		
O university hospital O academic teaching hospital O non-academic teaching hospital		
**Care level of the clinic**		
O basic care O standard care O priority care O maximum care		
**Catchment area**		
O rather urban O rather rural		
**Rank**		
O doctor in internschip O assistent in continuing education O assistent in specalist status O Senior physician in charge<br>O Senior physician / Head O Chief physician		
**Current job**	O yes O no	
**When working in a practice:**<br>(please check)		
**Academically connected practice?**		
O Teaching practice O unrelated practice		
**Organization type of practice**		
O practice without a partner O joint practice O practice sharing O medical care unit		
**Catchment area**		
O rather urban O rather rural		
**When working with partners or in a medical care unit**		
**Number of colleagues:**	O 1 O 2 O 3 O 4 O 5 O 6<br>O 7 O 8 O 9 O 10 O 11 O 12	
**Subjects (subject, specialization) of the practice colleagues:**		
**Current job**	O yes O no	

Attachment 1 to: *Zupanic M, Kreuer J, Bauer D, Nouns ZM, Ehlers JP, Fischer MR. Spontaneously retrievable knowledge of Germany general practitioners depending on time since graduation, measured with the progress test medicine.* GMS J Med Educ. 2020;37(5):Doc49. DOI: 10.3205/zma001342

Training/CME

**Training expenses in hours per normal working week**
Clinical activity (please enter below this line):
Own advanced training (please enter below this line):
Education and teaching (please enter below this line):
Research activities (please enter below this line):
**Use of different media in the CME**
**Textbooks**
○ rather 30 min/week ○ rather 60 min/week ○ over 60 min/week
**Journals**
○ rather 30 min/week ○ rather 60 min/week ○ over 60 min/week
**Training / congresses**
○ rather 30 min/week ○ rather 60 min/week ○ over 60 min/week
**Quality cicle**
○ rather 30 min/week ○ rather 60 min/week ○ over 60 min/week
**Balint groups**
○ rather 30 min/week ○ rather 60 min/week ○ over 60 min/week
**Consultation with colleagues**
○ rather 30 min/week ○ rather 60 min/week ○ over 60 min/week
**other (please enter below this line):**
○ rather 30 min/week ○ rather 60 min/week ○ over 60 min/week
**Thank you very much for the informations!**

Attachment 1 to: *Zupanic M, Kreuer J, Bauer D, Nouns ZM, Ehlers JP, Fischer MR. Spontaneously retrievable knowledge of Germany general practitioners depending on time since graduation, measured with the progress test medicine.* GMS J Med Educ. 2020;37(5):Doc49. DOI: 10.3205/zma001342
